# LEARNING FROM IN-DEPTH COGNITIVE ASSESSMENTS CONDUCTED IN RURAL INDEPENDENT LIVING FACILITIES

**DOI:** 10.1093/geroni/igz038.2040

**Published:** 2019-11-08

**Authors:** Lisa K Wiese, Christine Williams, James E Galvin, Debra Hain, David Newman

**Affiliations:** 1 C.E. Lynn College of Nursing, Florida Atlantic University, Boca Raton, United States; 2 Florida Atlantic University, Boca Raton, Florida, United States

## Abstract

Despite benefits to earlier Alzheimer’s disease interventions, efforts to increase dementia detection and treatment in high-risk rural areas are lacking. Barriers include lack of resources, including limited time for providers to conduct in-depth cognitive assessments. A pilot program tested the effectiveness of a home-based approach for increasing rates of AD detection and treatment in a rural retired farmworker community. Depression and cognitive screenings of 139 residents conducted by community health workers were followed up with in-depth geriatric-focused assessments, including the Moca-B, by experienced, culturally diverse adult gerontological nurse practitioners (AGNPs). Their findings were forwarded to primary providers. This approach was evaluated for effectiveness using correlations, regressions, and Chi-square analyses of variables on rates of ADRD screening, referrals, and ADRD diagnosis.

